# A High Spatiotemporal Iontronic Single-Cell Viscometer

**DOI:** 10.34133/2022/9859101

**Published:** 2022-06-29

**Authors:** Tianyang Zhang, Siyuan Yu, Bing Wang, Yitong Xu, Xiaomei Shi, Weiwei Zhao, Dechen Jiang, Hongyuan Chen, Jingjuan Xu

**Affiliations:** State Key Laboratory of Analytical Chemistry for Life Science, School of Chemistry and Chemical Engineering, Nanjing University, Nanjing 210023, China

## Abstract

Ideal single-cell viscometer has remained unachieved, leaving a gap in current palette of single-cell nanotools. Information of single-cell viscosity could contribute to our knowledge of fundamental biological processes, e.g., mass diffusion, biochemical interaction, and cellular responses to many diseases and pathologies. Although advances have been made to this end, existing methods generally suffer from limitations, e.g., low spatiotemporal resolution. Here, we describe a high spatiotemporal iontronic single-cell viscometer that operates upon a patch clamp integrated with double-barreled nanopores separated by a septum of ca. 32 nm. The system enables reversible electroosmotic manipulation of the adjacent small fluid bridging two nanopores, the viscous alternation of which could be sensitively monitored by the ionic responses. In practical cellular studies, significantly, our findings reveal not only the less deviated medium viscosities than those of lysosomes and mitochondria but also the highest viscosities in the near-nuclear region than those of mitochondrion-dense and lysosome-dense regions. This work has provided an accessible single-cell viscometer and enriched the armory of single-cell nanotools.

## 1. Introduction

Self-contained electrochemical nanotechnologies empowering direct single-cell studies have long been pursued to deepen our knowledge of cellular heterogeneity and behaviors as well as to promote precise pathological diagnosis and therapeutic strategies [ 1– 3]. Accessing the native cytosol by a transmembrane nanoelectrode is the prerequisite for subsequent electrochemical decipher of the hidden biomolecular information and intracellular dynamics [ 4, 5]. After penetration, application of potential-resolved electrochemistry at the conducting nanotips could trigger redox reactions that correlate with specific endogenous species, which is at the heart of current single-cell electroanalysis [ 6– 8]. Nevertheless, to yield electrical signals, occurrence of Faradic reactions at specific potentials and wired circuits are the necessity, which severely restricts this methodology to redox-involved events. To take it a step further, scientists have endeavored to exploit new nanoelectrochemistry and mechanism [ 9– 12]. For example, using a single nanopipette, confined electrochemiluminescence has recently been exploited for wireless intracellular analysis [ 9]. Photoelectrochemistry of engineered organic molecules and semiconductor hybrid has been used for studying intracellular oxidative stress [ 10]. Utilization of the ionic current rectification could even realize the detection of intracellular H _2_O _2_ [ 11] and microRNA [ 12].

Cell interior functions as a viscoelastic material, and cellular viscosity is crucial for the ubiquitous diffusion-dependent biological reactions and cascades [ 13, 14]. While changes in cellular viscosity has been correlated with many pathologies and malfunctions [ 15, 16], the homeostatic nature of cellular viscosity and the intrinsic viscosity regulation mechanism as well as its potential biochemical/biophysical implications are still not well understood. Caragine et al. recently reported observation of the movement and fusion of cellular components for investigation of the viscous properties of the cell [ 17]. Persson et al. then revealed that the cell tunes cytosolic viscosity to counter alternation of temperature and energy [ 18]. To perform these, studies demand the ability to measure viscosity directly in a living cell, which could provide a benchmark for monitoring the viscosity and identifying the impacts of specific diseases on the cellular material properties. Despite their respective advantages, existing methods, e.g., fluorescent and rotational magnetic ones [ 15, 17, 19, 20], are in concerns of low spatiotemporal resolution, biocompatibility, or bioorthogonality issues, such as possible cytotoxicity or destructiveness of the probe, disturbance of the intrinsic cellular chemistry, and their photobleaching or degradation inside cells. To date, scientists still lack an ideal single-cell viscometer that is highly simple, durable, and spatiotemporal-resolved.

Originally found in nature, iontronics has rapidly evolved as an advanced technology based on sophisticated control of ions as signal carriers, underpinning the operating rationale of aqueous circuits made of rationally designed nanostructures and thereon the controllable ionic transport [ 21– 23]. Working in aqueous environments, iontronic devices have shown to be promising for sensing, logic circuiting, and brain-machine interfacing. Herein, using the θ -type nanopores [ 24, 25], we report an accessible iontronic single-cell viscometer with a high spatiotemporal resolution (see Supporting Information for experimental details). The nanotool was operated upon the potential-directed reversible ionic motion within the nanoscale cytosolic fluid bridging two adjacent nanopores, which would create recordable ionic currents of nanoampere level. Using a theory that describes how viscosity affects the current, the altered cellular viscosity and differentiable signals could be linked, and the accurate viscosity at specific time and position could also be inferred. Glucose deprivation and heat shock experiments further revealed the practicability of this nanotool of high spatiotemporal resolution. This work achieves an accessible single-cell viscometer and sheds light on futuristic study of single-cell material property and assessment of associated chemotherapeutic effects.

## 2. Results

### 2.1. Operation Rationale and Measurement

**Figure 1:**
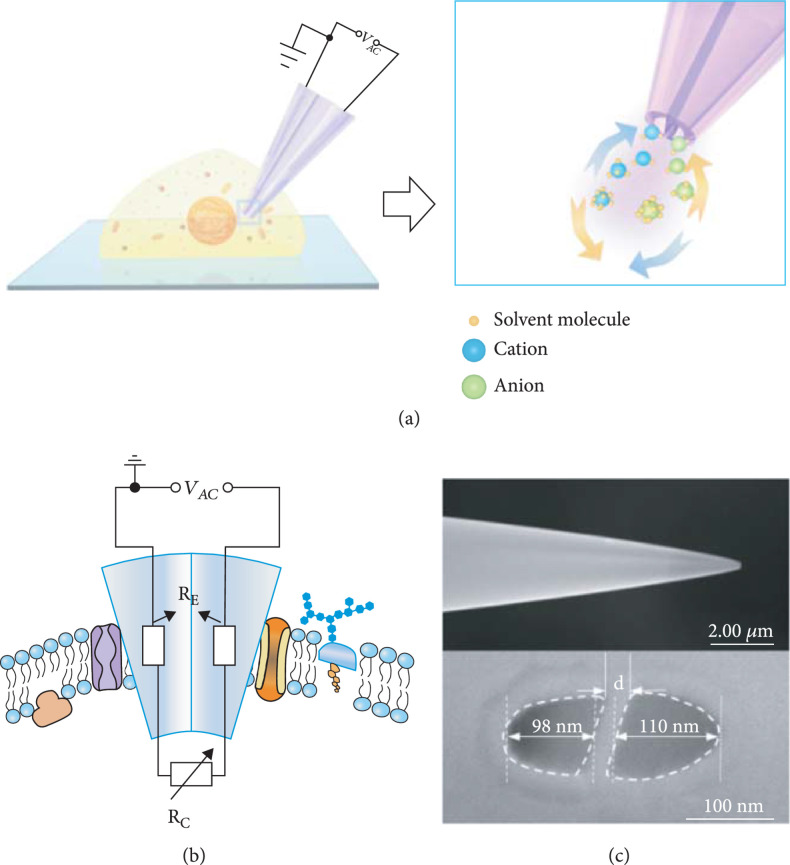
(a) Scheme of θ -type nanopore-based iontronic single-cell viscometer. (b) The corresponding equivalent circuit model. (c) The SEM side (upper) and top view (bottom) of the nanotool.

As illustrated in Figure 1(a), the nanotool was used to position at the specific intracellular site of a single living cell, and the local viscosity was transduced by the directional ionic motion driven by a step pulse voltage (SPV) from a patch-clamp system. Alternative application of the SPV (−1.0 V/+1.0 V) could induce reversible ionic motion passing through the two adjacent apertures, while the very short application time of ~400 ms was set to minimize the influence upon both the intracellular microenvironment and the interfacial charge property of the two lumens. According to the Nernst-Planck equation and Stokes-Einstein relation [ 26], the ionic diffusion coefficient relies closely upon the solution viscosity (Eqs. S1–S10), indicating that the ionic current is controlled by the medium viscosity. In the present case, as illustrated in Figure 1(b), the essential resistance (RE), consisting of those of nanopipettes, electrodes, and electrolyte, was kept unchanged throughout the study, whereas the resistance of the cytosol around the nanotips (RC) was the only variate during the measurement, suggesting the variation of the acquired ionic signal solely originated from the varied RC associated with the local viscosity. Figure 1(c) depicts the side-view (upper) and top-view (bottom) scanning electron microscopy (SEM) micrographs of the as-used laser-pulled double-barrel quartz capillaries with θ -type nanopores. The two compartmentalized semielliptical orifices possessed similar dimensions of 104±6nm , which were separated by a quartz septum of 32±3nm in width. Incidentally, to ensure better performance, similar devices were preferred, which could be easily screened via the SPV-induced ionic responses, as reflected in and discussed with Figures S1–S2, respectively.

**Figure 2:**
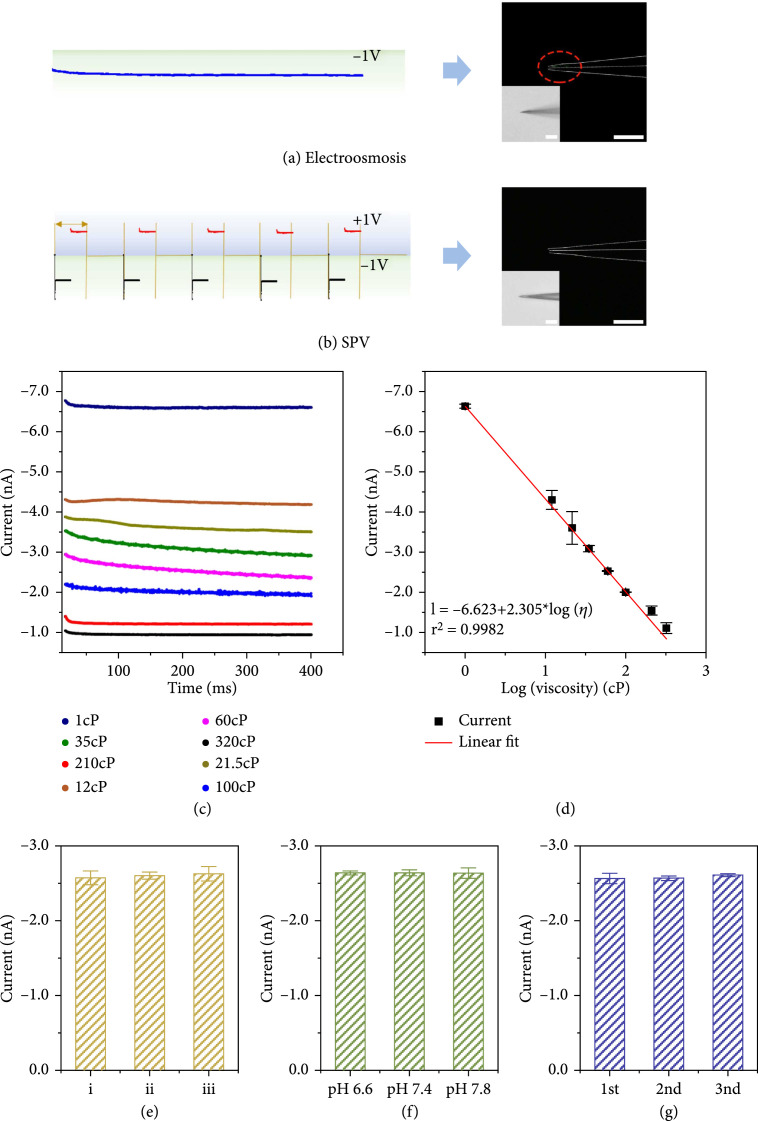
(a) The electroosmotic effect (20 min) induced the presence of fluorescein within the lumen. Scalebar=5μm . (b) SPV caused no appearance of the fluorescence. (c) Ionic responses in glycerin-HEPES milieus with different viscosities under -1.0 V. (d) The corresponding derived linearity of log viscosity (cP) vs. ionic signals (nA). (e) The ionic effects of (i) 2.5 mM, (ii) 10 mM, and (iii) 15 mM Na ^+^. (f) The pH values of 6.6, 7.4, and 7.8 in HEPES milieu of 60 cP. (g) Three adsorption tests were sequentially performed after immersing the nanotool in the cell lysate for 10 min.

The practical applicability of the nanotool was then in vitro studied. Previous reports had revealed the potential-directed delivery or sampling functions of nanopipettes [ 2, 10, 27, 28], the function of which was initially investigated using HEPES milieu containing saturated fluorescein. As shown in Figure 2(a), such an effect was verified by 20 min electroosmosis under -1.0 V, which led to obvious green fluorescence of fluorescein within the nanotool. By contrast, as shown in Figure 2(b), the SPV test under the same conditions could not induce the appearance of the fluorescence, indicating the absence of fluorescein within the nanotool. This was due to the very short SPV time, i.e., 400 ms, and the rapid application of inverse voltage. Next, the responses of the nanotool were acquired in specific glycerin-HEPES milieus of different viscosities ranging from 1.0 to 320 cP (Table S1), which were set in advance with the assistance of a commercial viscometer. As shown by the differentiable i - t curves in Figure 2(c), the generated ionic signals were gradually inhibited from ca. 6.7 to ca. 1.0 nA, which could be attributed to the reduced ionic diffusion coefficient with the increase in viscosity. Figure 2(d) shows the derived linear relationship between the ionic signals and the corresponding viscosities. Incidentally, the effects of possible ionic variation (Figure 2(e)), pH change (Figure 2(f)), and adsorption (Figure 2(g)) were also excluded. As shown in Figure 2(e), the HEPES milieu of 60 cP containing 130 mM K ^+^ and different Na ^+^ concentrations of (i) 2.5 mM, (ii) 10 mM, and (iii) 15 mM were tested, and hardly any signal variation could be observed. Besides, to study the possible pH variation effect, as shown in Figure 2(f), HEPES milieu of 60 cP with pH of 6.6, 7.4, and 7.8 had almost no effect on the response of the nanotool [ 10, 14]. To study the possible adsorption effect, the nanotool was sequentially immersed into the cell lysate for 10 min and then tested for three repeated times. As shown in Figure 2(g), the nearly identical signals indicated that there was little adsorption on the nanotool. Besides, the small droplet experiments further confirmed not only its capability to distinguish different viscosities but also its stability during the repeated measurements (Figure S3).

### 2.2. Cell Spatial-Resolved Measurement

**Figure 3:**
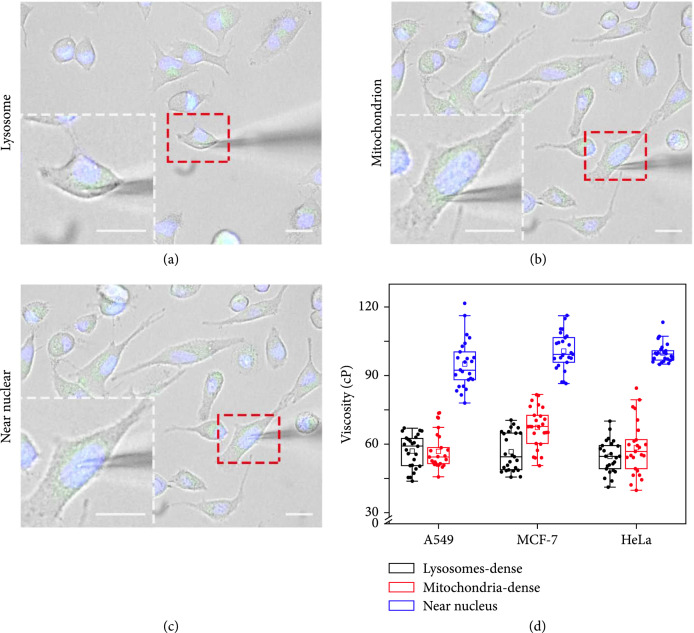
(a) Merged microscopy images of controlling the nanotool to the lysosome-dense, mitochondria-dense, and nuclear periphery regions in representative A549 cells. Scalebar=10μm . (b) The corresponding viscosities at these subcellular regions ( n=25 ).

Its capability for in vivo spatial-resolved studies was then studied through targeting specific subcellular regions. Exemplified by three individual A549, MCF-7, and HeLa cells, three specific subcellular regions, i.e., the lysosome-dense, mitochondrion-dense, and the near-nuclear ones, were chosen. As shown in Figures S4–S6, it was observed that the penetration into the cytoplasm by the nanotool could not induce the change of the cellular morphologies. Our earlier staining studies had indicated the minimal destructiveness of such nanotools without affecting the cytomembrane integrity and cellular viability [ 11, 12], which was also confirmed in the present case of double-barreled nanopores (Figures S7–S11). Three different dyes, i.e., Lyso-Tracker Green, MitoGreen, and Hoechst 33342, were used to locate lysosomes, mitochondria, and nucleus, respectively. The abovementioned three subcellular locations of three different cells were then targeted as represented in Figures 3(a)– 3(c) and Figures S12–S14, with 25 cells per group. The corresponding current during the nanotip penetrated throughout the cell membrane

As shown in Figure S15 and Figure 3(d), the ionic responses of these three positions could be, respectively, measured with the derived average viscosities of ca. 58 cP, 55 cP, and 93 cP for A549 cells, of ca. 55 cP, 68 cP, and 99 cP for MCF-7 cells, and of ca. 55 cP, 57 cP, and 99 cP for HeLa cells. Although significantly biased lysosomal viscosities of ca. 65-130 cP [ 29, 30] and mitochondrial viscosities of ca. 62-120 cP [ 15, 30– 32] had been reported by respective optical methods, the medium viscosities in the mitochondrion-dense and lysosome-dense regions are largely unknown. Our statistical results revealed the less deviated viscosities around these organelles. In particular, as compared to those of the two subcellular regions, our results further disclosed the highest viscosities ranging from ca. 80 to 130 cP in the near-nuclear region. This phenomenon may be due to the dense distribution of the cytoskeleton next to the nucleus. Tubulin, the main component of the cytoskeleton, has been reported to have great influence on the local cytoplasmic viscosity [ 26, 33, 34].

### 2.3. Cell Temporal-Resolved Measurement

**Figure 4:**
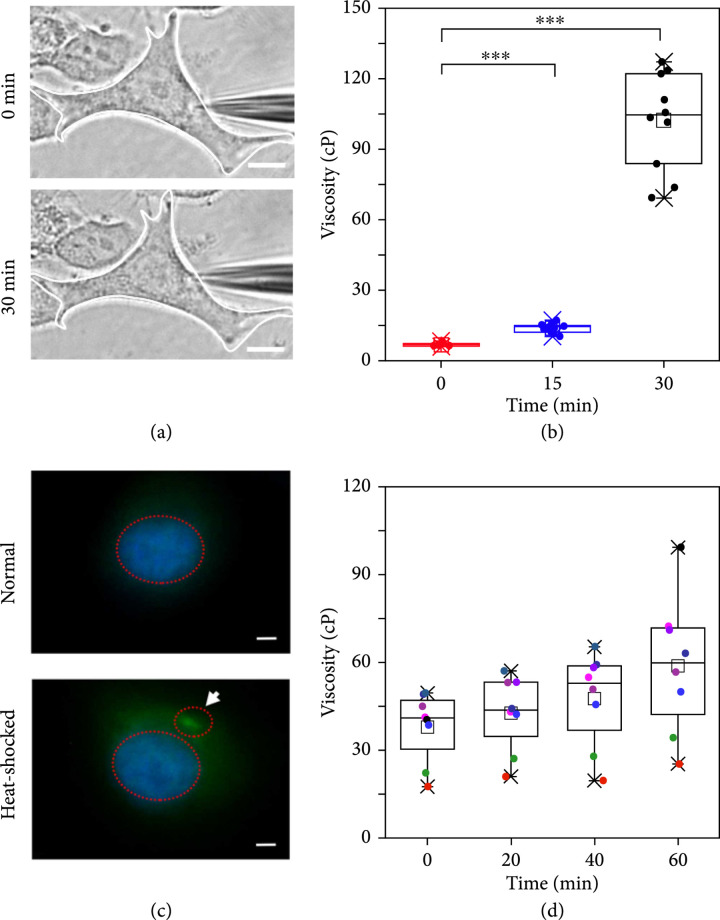
(a) Bright-field microscopy images of the nanotool recording the ionic signals within the single HeLa cell upon glucose deprivation experiment. Scalebar=10μm . (b) The measured viscosities ( n=10 , p<0.001 ). (c) The fluorescence images of normal and heat-shocked HeLa cells labeled with TIA-1-GFP as SG markers. Scalebar=10μm . (d) The recovery of the viscosities of heat-shocked HeLa cells within 60 minutes ( n=8 ).

The nanotool was then implemented for in vitro temporal-resolved studies via subjecting the representative HeLa cells to glucose deprivation and heat shock experiments, respectively. Intracellular deprivation of glucose was initially performed to induce the enhanced cytoplasmic viscosity [ 18]. Experimentally, after 12 h culture in glucose-containing Dulbecco’s modified Eagle medium (DMEM), the HeLa cells were treated with glucose-free DMEM for 30 min for glucose deprivation, followed by collection of the ionic responses every 15 minutes within the cytoplasm. As shown in Figure 4(a), the targeted cell well maintained its morphology during the 30 min detection. As compared to the case of normal HeLa cells with the viscosity fluctuation around ca. 60 cP (Figure S16), Figure 4(b) records the obvious varied viscosity responses with increase from ca. 7 to 106 cP with 10 treated HeLa cells per group [ 18].

Stress granules (SGs) are a membraneless organelle composed of protein-wrapped RNA, which undergo a nucleated assembly mechanism under stress conditions (like oxidative stress, heat shock, and ultraviolet irradiation) [ 35, 36]. Formation of SGs is closely related to many diseases, e.g., tumor apoptosis and Alzheimer’s disease; hence, exploration of SG-associated physiological indexes is of great significance [ 37– 39]. Heat shock experiment was then conducted with treatment at 42°C for 70 minutes and then 37°C for 30 minutes to induce the formation of SGs [ 18, 40], which was proven via an indirect immunofluorescence assay, with the assistance of Hoechst 33342 to stain the nucleus (Figure S17). As compared in Figure 4(c), no green fluorescence in the normal HeLa cell indicated the absence of SGs in the cytoplasm, whereas the appearance of green fluorescence around the blue nucleus indicated the assembly of intracellular SGs (white arrow). After 60 min recovery, the disappearance of the green granules indicated the disassembly of SGs [ 18, 37– 40]. As shown in Figure 4(d) and Figure S18, decreased viscosity of ca. 40 cP was measured after the formation of SGs from heat shock. With the increase in time, the increase in the viscosity was gradually observed and recovered to normal level of ca. 60 cP within 60 minutes. This phenomenon could be attributed to the removal of stress conditions and thus the depolymerization of SGs, which was also confirmed with dark field observation. As recorded in Figure S19, there were many bright particles in the heat-shocked cells, which then gradually disappeared within the same 60 minutes.

## 3. Discussion

To conclude, we have devised a high spatiotemporal iontronic single-cell viscometer based on the potential-controlled electroosmotic manipulation of ionic flow confined within the θ -type nanopores. The miniaturized θ -type nanotip permits cytomembrane penetration under physiological conditions with cell-context preservation and allows the reversible ionic motion within the two adjacent nanopores; the generation of the varied ionic currents could be intimately correlated with the altered intracellular viscosities. Significantly, practical spatial studies disclosed not only the less deviated medium viscosities of the subcellular lysosome- and mitochondria-dense regions than those of the organelles themselves but also the highest viscosities in the near-nuclear region among the studied three subregions. Exemplified by the glucose deprivation and heat shock experiments, changes of cellular viscosity were further temporally revealed. As compared to current implementations of θ -type nanopores (Table S2), this work presented a new application scenario, which should potentially contribute to the futuristic investigation of single-cell viscosity, single-cell diagnostics, and assessment of specific drugs and chemotherapies.

## 4. Materials and Methods

### 4.1. Reagents and Materials

Sodium chloride (NaCl), potassium chloride (KCl), magnesium chloride (MgCl _2_), glycerin, calcium chloride anhydrous (CaCl _2_), 2-[4-(2-hydroxyethyl) piperazin-1-yl] ethanesulfonic acid (HEPES), fluorescein, paraformaldehyde (POM), Tween-20, silver wire ( Φ 0.2 mm), and other reagents were purchased from Sinopharm Co., Ltd. (Shanghai, China). Bovine serum albumin (BSA) was purchased from Sigma-Aldrich (St. Louis, MO, USA) (Sigma). All aqueous solutions were prepared in deionized water (Millipore) with a resistivity of 18.2MΩ∗cm . 1x PBS (phosphate-buffered solution, 10 mM, pH 7.2-7.4), Triton X-100, 2.2 mM EDTA solution containing 0.25% trypsin, Hoechst 33342, propidium iodide (PI), MitoGreen, HeLa cells, A549, and MCF-7 cells were purchased from Keygenbio Co., Ltd. (Nanjing, China). Lyso-Tracker Green was from Beyotime Biotechnology (China). Fetal Bovine Serum (FBS), DMEM, and normal RPMI 1640 medium were purchased from Gibco (USA). Rabbit monoclonal anti-TIA-1 antibody [EPR9304] AB 140595 and goat anti-rabbit secondary antiserum lgG H&L (Alexa Fluor® 488) ab 150077 were both obtained from Abcam (USA).

### 4.2. Experimental Setup and Data Acquisition

All ionic current measurements were recorded using a Multiclamp 700B amplifier (Axon Instruments, USA) in voltage-clamp mode with the Digidata 1550 digitizer (Molecular Devices) and a PC equipped with pCLAMP10.5 software (Molecular Devices). The current-voltage ( I - V ) curves were recorded by sweeping the voltage from -1.0 V to +1.0 V and recorded with a sampling frequency of 5 kHz. A three-dimensional MP-225 micromanipulator (Sutter Instrument, Novato, CA) equipped with an inverted microscope (Ti2-E, Nikon, Japan) was applied for the precise control of the nanopipette to insert into cells under observation. The I - V and I - t recordings were plotted with Clampfit 10.5 software and OriginLab. Scanning electron microscopic (SEM) characterization was performed on a JSM-7800F instrument (JEOL, Japan), equipped with Stage Top Incubator (STX-EN-01, Tokai Hit Co., Ltd).

### 4.3. Fabrication of the Nanodevice

Quartz theta (O.D.: 1.2 mm, I.D.: 0.90 mm; 7.5 cm length) was purchased from Sutter Instrument and were laser-pulled by using a P-2000 pipette puller (Sutter Instrument, Novato, CA, USA) with a two-line program containing the following parameters: line 1: heat=900 , Fil=4 , Vel=30 , Del=180 , and pull=40 , and line 2: heat=950 , Fil=3 , Vel=20 , Del=180 , and pull=120 . To ensure the reproducibility of nanotip geometry, the variation of pulling time was controlled within 0.2 second. Prior to electrochemical viscosity measurement, silver wires were immersed into the commercially 84 disinfectant for 25 min at room temperature to fabricate home-made Ag/AgCl wire electrodes. Then, two Ag/AgCl electrodes were, respectively, served as the working electrode and counter electrode as well as reference electrode inserted into two pores of the θ -nanopipette, which were then backfilled with NaCl 5 mM, KCl 120 mM, MgCl _2_ 4.5 mM, and HEPES 10 mM ( pH=7.4 ).

### 4.4. Cell Culture

HeLa cells and A549 cells were cultured with DMEM in the presence of 10% FBS and antibiotics (penicillin and streptomycin) and maintained at 37°C in 5% CO _2_/95% air, while MCF-7 cells were cultured with normal RPMI 1640 medium in the presence of 10% FBS and antibiotics (penicillin and streptomycin) and maintained at 37°C in 5% CO _2_/95% air.

### 4.5. Cell Lysate

HeLa cells were digested using the trypsin enzyme and then centrifuged with 1000 rpm for 5 min. Next, the sediment was diluted with 1 ml 1X PBS, and the cell density was then calculated via countess II (Life, the USA). Finally, the cell lysate was obtained by treatment with the diluted cell solution at 0°C using an ultrasonic cell crusher noise isolating chamber (Anxiu, China), with the program as follows: 35% power, 2 min and rod 6, and ultrasound performed for 5 s and paused for 5 s.

### 4.6. Cell Vitality Measurement

The cellular vitality tests were performed via the dye staining experiments with the usage of propidium iodide (PI) and Hoechst 33342 (Figures S7–S10), respectively.

### 4.7. Fluorescence Observation

To locate lysosomes, Lyso-Tracker Green was diluted to 1/9250 ( V/V ) with DMEM, and A549, MCF-7, and HeLa cells were washed three times with 1x PBS. Next, the cells were incubated in the diluted Lyso-Tracker Green solution at room temperature for 30 minutes. To locate mitochondria, MitoGreen was diluted to 1/1000 ( V/V ) with PBS, and A549, MCF-7, and HeLa cells were washed three times with 1x PBS. Next, the cells were incubated in the diluted MitoGreen solution at room temperature for 15 minutes. To locate the nucleus, the cells were washed three times with 1x PBS and then stained with Hoechst 33342 at room temperature for 10 min (Figures S11–S13).

### 4.8. Characterization of the Formation of Intracellular Stress Granules

TIA-1 is an RNA-binding protein, which is regarded as playing a key role in SG assembly [ 41]. Thus, SG degradation is able to be observed via tracking the statement of TIA-1 when the stress conditions alleviated. Experimentally, HeLa cells growing on the cell culture dish were fixed with 4% paraformaldehyde in PBS for 10 min at room temperature and then permeabilized with 0.3% TritonX-100 for 10 min, followed by incubation in blocking buffer (5% BSA and 0.1% Tween-20 in PBS) for 1 h before the addition of primary antibodies. A primary antibody of rabbit monoclonal anti-TIA-1 and goat anti-rabbit secondary antiserum lgG H&L were, respectively, diluted to 1 : 300 ( V/V ) and 1 : 1000 ( V/V ) using the blocking buffer. To characterize the stress granules, the heat-shocked HeLa cells were incubated in the diluted primary antibody solution at 4°C overnight. Then, after washing several times in PBS, the cells were incubated in diluted solution of the secondary antibody for 1 h in the dark. Then, the cells were rinsed three times with PBS and stained with Hoechst 33342 for 5 min to locate the nucleus (Figure S16).

### 4.9. Observation of Heat-Shocked Cells under Dark Field

Heat-shocked HeLa cells (42°C for 70 minutes and 37°C for 30 minutes) were observed and photographed by inverted microscopy (IX71, Olympus) coupled with a true-color digital camera (Olympus DP80, Japan) in 10% FBS 1x PBS solution (Figure S17).

## Data Availability

All data are available in the main text or supplementary materials. All other relevant source data are available from the corresponding authors upon reasonable request.

## Supplementary Materials

Supplementary Materials Figure S1: (a) the SPV-induced ionic response at +1.0 V (blue curve), 0 V (red curve), and -1.0 V (black curve) in 1 cP solution. (b) The stability of the ionic current under -1.0 V in 60 cP solution for 20 min. Figure S2: (a) ionic currents of nine as-screened nanotools under -1.0 V and+ 1.0 V. (b) The corresponding $I_{-1.0\text{V}}/{\text{I}}_{+1.0\text{V}}$ values. Table S1: the glycerin-HEPES mixtures (10 mM NaCl, 130 mM KCl, 4.5 mM MgCl _2_, and 10 mM HEPES, pH=7.4 ) of different viscosities at 20°C. Figure S3: (a) experimental setup of the small droplet detection. (b) Corresponding current signals of small droplet detection in 1 cP, 60 cP, and 100 cP solutions. Figure S4: bright-field images of A549 cells before and after penetration and withdrawal of the nanopipette. Scale bar: 50 *μ*m. Figure S5: bright-field images of MCF-7 cells before and after penetration and withdrawal of the nanopipette. Scale bar: 50 *μ*m. Figure S6: bright-field images of HeLa cells before and after penetration and withdrawal of the nanopipette. Scale bar: 50 *μ*m. Figure S7: fluorescent and merged images of PI-stained HeLa cells before and after the penetration and withdrawal of the nanotool. Scale bar: 50 *μ*m. Figure S8: fluorescent and merged images of Hoechst 33342-stained HeLa cells before and after the penetration and withdrawal of the nanotool. Scale bar: 50 *μ*m. Figure S9: fluorescent and merged images of PI-stained MCF-10A cells before and after the penetration and withdrawal of the nanotool. Scale bar: 25 *μ*m. Figure S10: fluorescent and merged images of Hoechst 33342-stained MCF-10A cells before and after the penetration and withdrawal of the nanotool. Scale bar: 25 *μ*m. Figure S11: the current change of the nanotool during its insertion and withdrawal from the cell membrane at - 200 mV. Figure S12: in vivo spatial-resolved location of the mitochondrion-dense, lysosome-dense, and near-nuclear regions of specific A549 cells with the assistance of Lyso-Tracker Green, MitoGreen, and Hoechst 33342 dyes. Scale bar: 50 *μ*m. Figure S13: in vivo spatial-resolved location of the mitochondrion-dense, lysosome-dense, and near-nuclear regions of specific MCF-7 cells with the assistance of Lyso-Tracker Green, MitoGreen, and Hoechst 33342 dyes. Scale bar: 50 *μ*m. Figure S14: in vivo spatial-resolved location of the mitochondrion-dense, lysosome-dense, and near-nuclear regions of specific HeLa cells with the assistance of Lyso-Tracker Green, MitoGreen, and Hoechst 33342 dyes. Scale bar: 50 *μ*m. Figure S15: the corresponding ionic currents collected at lysosome-dense, mitochondrion-dense, and near-nucleus regions ( n=25 ). Figure S16: viscosity of normal HeLa cells within 60 minutes. Figure S17: fluorescence microscopy images of TIA-1-marked normal, heat-shocked, and recovered HeLa cells after 60-minute recovery. Scale bar: 10 *μ*m. Figure S18: (a) bright-field microscopy images of the nanotool recording the ionic signals within the single HeLa cell upon heat shock recovery experiment. Scalebar=10μm . Figure S19: (a) dark field images of the heat-shocked HeLa cell during 100-minute observation. (b) The corresponding change of the light intensities. Scalebar=20μm . Table S2: some typical applications of θ -nanopipette in bioanalysis and beyond.
