# Supplementary material for: A High Spatiotemporal Iontronic Single-Cell Viscometer
Source: Research (Wash D C). 2022 Jun 29;2022:9859101. doi: 10.34133/2022/9859101 (PMC11697695; doi:10.34133/2022/9859101)
Supplement: Supplementary Materials — Figure S1: (a) the SPV-induced ionic response at +1.0 V (blue curve), 0 V (red curve), and -1.0 V (black curve) in 1 cP solution. (b) The stability of the ionic current under -1.0 V in 60 cP solution for 20 min. Figure S2: (a) ionic currents of nine as-screened nanotools under -1.0 V and+ 1.0 V. (b) The corresponding I−1.0V/I+1.0V values. Table S1: the glycerin-HEPES mixtures (10 mM NaCl, 130 mM KCl, 4.5 mM MgCl 2, and 10 mM HEPES, pH=7.4 ) of different viscosities at 20°C. Figure S3: (a) experimental setup of the small droplet detection. (b) Corresponding current signals of small droplet detection in 1 cP, 60 cP, and 100 cP solutions. Figure S4: bright-field images of A549 cells before and after penetration and withdrawal of the nanopipette. Scale bar: 50 μm. Figure S5: bright-field images of MCF-7 cells before and after penetration and withdrawal of the nanopipette. Scale bar: 50 μm. Figure S6: bright-field images of HeLa cells before and after penetration and withdrawal of the nanopipette. Scale bar: 50 μm. Figure S7: fluorescent and merged images of PI-stained HeLa cells before and after the penetration and withdrawal of the nanotool. Scale bar: 50 μm. Figure S8: fluorescent and merged images of Hoechst 33342-stained HeLa cells before and after the penetration and withdrawal of the nanotool. Scale bar: 50 μm. Figure S9: fluorescent and merged images of PI-stained MCF-10A cells before and after the penetration and withdrawal of the nanotool. Scale bar: 25 μm. Figure S10: fluorescent and merged images of Hoechst 33342-stained MCF-10A cells before and after the penetration and withdrawal of the nanotool. Scale bar: 25 μm. Figure S11: the current change of the nanotool during its insertion and withdrawal from the cell membrane at - 200 mV. Figure S12: in vivo spatial-resolved location of the mitochondrion-dense, lysosome-dense, and near-nuclear regions of specific A549 cells with the assistance of Lyso-Tracker Green, MitoGreen, and Hoechst 33342 dyes. Scale bar: 50 [file 9859101.f1.docx]

Supplementary Materials for

A High Spatiotemporal Iontronic Single-Cell Viscometer

Tianyang Zhang^1†^, Siyuan Yu^1†^, Bing Wang^1^, Yitong Xu^1^, Xiaomei Shi^1^, Weiwei Zhao^1^*, Dechen Jiang^1^, Hongyuan Chen^1^, and Jingjuan Xu^1^*

1. State Key Laboratory of Analytical Chemistry for Life Science, School of Chemistry and Chemical Engineering, Nanjing University, Nanjing 210023 (China)

Correspondence should be addressed to Wei-Wei Zhao; zww@nju.edu.cn and Jing-Juan Xu; [xujj@nju.edu.cn](mailto:xujj@nju.edu.cn)

[^†^] These authors contributed equally to this work.

Content of Supplementary Materials

Text ST1. Operation Rationale.

Figure S1. a) The SPV-induced ionic response at +1.0 V (blue curve), 0 V (red curve) and -1.0 V (black curve) in 1 cP solution. b) The stability of the ionic current under -1.0 V in 60 cP solution for 20 min.

Figure S2. a) Ionic currents of nine as-screened nanotools under -1.0 V and +1.0 V. b) The corresponding I_-1.0V_/I_+1.0v_ values.

Table S1. The glycerin-HEPES mixtures (10 mM NaCl, 130 mM KCl, 4.5 mM MgCl_2_, 10 mM HEPES, pH=7.4) of different viscosities at 20 °C.

Figure S3**.** a) Experimental setup of the small droplet detection. b) Corresponding current signals of small droplet detection in 1 cP, 60 cP and 100 cP solutions.

Figure S4. Bright-field images of A549 cells before and after penetration and withdrawal of the nanopipette. Scale bar: 50 μm.

Figure S5**.** Bright-field images of MCF-7 cells before and after penetration and withdrawal of the nanopipette. Scale bar: 50 μm.

Figure S6. Bright-field images of HeLa cells before and after penetration and withdrawal of the nanopipette. Scale bar: 50 μm.

Figure S7. Fluorescent and merged images of PI stained HeLa cells before and after the penetration and withdrawal of the nanotool. Scale bar: 50 μm.

Figure S8. Fluorescent and merged images of Hoechst 33342 stained HeLa cells before and after the penetration and withdrawal of the nanotool. Scale bar: 50 μm.

Figure S9. Fluorescent and merged images of PI stained MCF-10A cells before and after the penetration and withdrawal of the nanotool. Scale bar: 25 μm.

Figure S10. Fluorescent and merged images of Hoechst 33342 stained MCF-10A cells before and after the penetration and withdrawal of the nanotool. Scale bar: 25 μm.

Figure S11: The current change of the nanotool during its inserting and withdrawing from the cell membrane at - 200 mV.

Figure S12**.** In vivo spatial-resolved location of the mitochondrion-dense, lysosome-dense and near nuclear regions of specific A549 cells with the assistance of Lyso-Tracker Green, MitoGreen and Hoechst 33342 dyes. Scale bar: 50 μm.

Figure S13. In vivo spatial-resolved location of the mitochondrion-dense, lysosome-dense and near nuclear regions of specific MCF-7 cells with the assistance of Lyso-Tracker Green, MitoGreen and Hoechst 33342 dyes. Scale bar: 50 μm.

Figure S14. In vivo spatial-resolved location of the mitochondrion-dense, lysosome-dense and near nuclear regions of specific HeLa cells with the assistance of Lyso-Tracker Green, MitoGreen and Hoechst 33342 dyes. Scale bar: 50 μm.

Figure S15. The corresponding ionic currents collected at lysosomes-dense, mitochondria-dense, and near-nucleus regions (n=25).

Figure S16. Viscosity of normal HeLa cell within 60 minutes.

Figure S17. Fluorescence microscopy images of TIA-1 marked normal, heat shocked and recovered HeLa cells after 60 minutes recovery. Scale bar: 10 μm.

Figure S18. a) Bright-field microscopy images of the nanotool recording the ionic signals within the single HeLa cell upon heat-shock recovery experiment. Scale bar = 10 μm.

Figure S19. a) Dark field images of the heat-shocked HeLa cell during 100 minutes observation; b) The corresponding change of the light intensities. Scale bar = 20 μm.

Table S2. Some typical applications of θ-nanopipette in bioanalysis and beyond.

**Supplementary Information Text**

**Text ST1. Operation Rationale**

The electric field produced by extra applied voltage as well as the electrical double layer (EDL) of surface could intrigue the movement of ions and the advection of solvent, the viscosity of which would affect the movement speed of the charged species according to the Nernst-Planck equation:^[1-2]^

$\text{J}_{\text{i}}\left( \text{x} \right)\text{=-}\text{D}_{\text{i}}\text{(}\text{∇}\text{c}_{\text{i}}\text{+}\frac{\text{F}}{\text{RT}}\text{c}_{\text{i}}\text{z}_{\text{i}}\text{∇}\text{φ)}$ (s1)

In the equation,$\text{ }\text{J}_{\text{i}}$*:* the flow of ion *i*(mol s^-1^cm^-2^), *x*: the distance, cm, $\text{D}_{\text{i}}$*:* the ionic diffusion coefficient of i(cm^2^s^-1^), *c_i_*: the concentration of *I* (mol cm^-3^), *F*: Faraday constant, 96485C/mol, *R*: Gas constant, 8.314J/(mol*K), *T*: temperature, K, *z_i_* : the valence of *i*, *φ*: potential, *V*. When i are specific ions, the *J_i_*(x) represents the current density corresponding to i at the position x. In another word, the current would only in connection with the ionic diffusion coefficient while the ceteris paribus. Considering the previous statement, Stokes-Einstein (SE) relation and Debye-Stokes-Einstein (DSE) relation are then added as the constraint condition, which are listed as followed.

$\frac{\text{Dτ}}{\text{T}}\text{=c}$ (s2)

$\frac{\text{D}_{\text{r}}\text{τ}}{\text{T}}\text{=}\text{c}_{\text{r}}$ (s3)

In these relations, *T* is the temperature, and the constants *c* and *c_r_* depend on the geometry of the ions and the boundary conditions. *τ* is a relaxation time proportional to the viscosity of the liquid, which can be calculated by *T_c_*, the time associated with molecular rotation, which follows:

$\text{T}_{\text{C}}\text{=}\frac{\text{1}}{\text{D}_{\text{r}}}\text{=}\text{-}\frac{\text{4π}\text{a}^{\text{3}}\text{η}}{\text{3kT}}$ (s4)

In these relations, *a* is the molecular radius, cm, *η* is the viscosity of the liquid, mPa‧s, *k* is Boltzmann constant, 1.380649 × 10^-23^ J/K, when the viscosity of the liquid is lower than 2700 cP, the movement of molecules follows narrowing movement condition (ω_0_T_c_ ≪ 1, ω_0_ is Lamar frequency), leading to:

$\text{J}_{\text{i}}\left( \text{x} \right)\text{=-}\frac{\text{3kT}}{\text{4π}\text{a}^{\text{3}}\text{η}}\text{(}\text{∇}\text{c}_{\text{i}}\text{+}\frac{\text{F}}{\text{RT}}\text{c}_{\text{i}}\text{z}_{\text{i}}\text{∇}\text{φ)}$ (s5)

Hence the SE and DSE relations provide a simple connection between mass and momentum transport in a liquid, validating that the current is linearly related to the viscosity of the solution in certain conditions.

According to the paper, ^[3]^ the power density of current pulsations *S_l_(ω)*, the connection has the form

$S_{l}\left( \omega, L,H \right) = p_{\alpha\beta}(\omega,L)\times\int_{-H}^{H} d\left( z-z_{1} \right)W_{\alpha\beta}(\omega, z-z_{1})(H-\left| z-z_{1} \right|)$ (s6)

$W_{\alpha\beta}\left( \omega, z-z_{1} \right)=\text{J}_{\text{i}}\left( \text{x} \right)/V$ (s7)

Here H is the z-dimension of electrode, ω is the random frequency, indexes α and β: equal x/y. The functions Wαβ are related to velocity correlators. V is the unit flow volume, microelectrode current pulsations is equal to the product of transfer functions pαβ and the integral of velocity correlators Wαβ in unit flow volume,$\int_{-H}^{H} d\left( z-z_{1} \right)(H-\left| z-z_{1} \right|)$. In other words, the product of transfer functions pαβ and the integral of unit flow. Thus, the following equality holds

$S_{l}\left( \omega, L,H \right) = p_{\alpha\beta}\left( \omega,L \right)\times\int d(J)$ (s8)

Combining equation s5 and s8,

$S_{l}\left( \omega, L,H \right) = \text{-}\frac{\text{3kT}}{\text{4π}\text{a}^{\text{3}}}p_{\alpha\beta}\left( \omega,L \right)\text{(}\text{∇}\text{c}_{\text{i}}\text{+}\frac{\text{F}}{\text{RT}}\text{c}_{\text{i}}\text{z}_{\text{i}}\text{∇}\text{φ)}\ln\text{η}$ (s9)

Considering the resistance and other current loss conditions,

$S_{l}\left( \omega, L,H \right) = \text{-}\frac{\text{3kT}}{\text{4π}\text{a}^{\text{3}}}p_{\alpha\beta}\left( \omega,L \right)\text{(}\text{∇}\text{c}_{\text{i}}\text{+}\frac{\text{F}}{\text{RT}}\text{c}_{\text{i}}\text{z}_{\text{i}}\text{∇}\text{φ)}\text{×}\text{2.303} \log\eta\text{+}\text{c}$ (s10)

Here, C is constants.

As shown in Equation S10, the ionic current is proportionate to the logarithm of viscosity, which is consistent with our experimental data.

Stability and Reproducibility of the Viscometer.


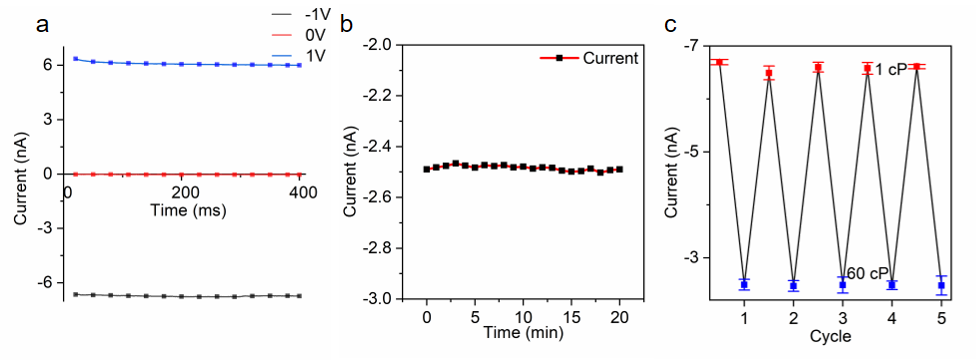


Figure S1. a) The SPV-induced ionic response at +1.0 V (blue curve), 0 V (red curve) and -1.0 V (black curve) in 1 cP solution. b) The stability of the ionic current under -1.0 V in 60 cP solution for 20 min. c) The ionic current under -1.0 V of the nanopipettes in 1 cP solution and 60 cP solution for 5 cycles

Figure S1a shows the SPV-induced ionic response of the as-fabricated nanotool in 1 cP solution. With the respective application of +1.0 V, 0 V and -1.0 V, differentiable ionic currents could be stably produced within the voltage applied time of 400 ms. Figure S1b and Figure S1c shows the characterization of the nanopipette reproducibility.


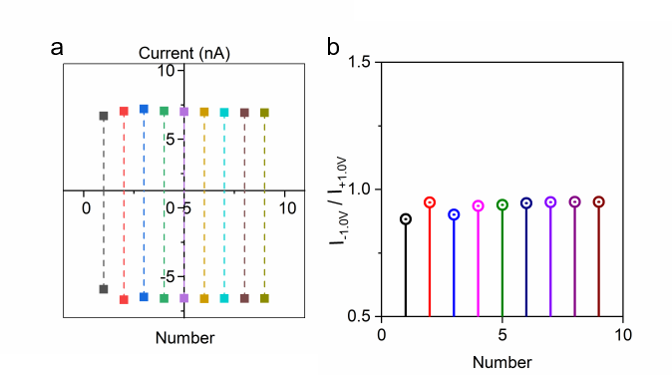


Figure S2. a) Ionic currents of nine as-screened nanotools under -1.0 V and +1.0 V. b) The corresponding I_-1.0V_/I_+1.0v_ values.

Figure S2a shows the SPV-induced ionic currents at +1.0 V and -1.0 V of nine as-screened nanotools and Figure S2b shows the corresponding I_-1.0 V_/I_+1.0 V_ values. Only those with identical responses were kept for further experiments, others with larger deviations were discarded and not used for the cellular application.

Preparation of Solutions with Viscosities Ranging from 1.0 to 320 cP.

Table S1**.** The glycerin-HEPES mixtures (10 mM NaCl, 130 mM KCl, 4.5 mM MgCl_2_, 10 mM HEPES, pH=7.4) of different viscosities at 20 °C.

| Water (v%) | Glycerin (v%) | Viscosity (cP) |
| --- | --- | --- |
| 100 | 0 | 1 |
| 38 | 62 | 12 |
| 35 | 65 | 21.5 |
| 30 | 70 | 35 |
| 25 | 75 | 60 |
| 18 | 82 | 100 |
| 12 | 88 | 210 |
| 9 | 91 | 320 |

The mixture solution (pH=7.4) containing 10 mM NaCl, 130 mM KCl, 4.5 mM MgCl_2_, 10 mM HEPES and various proportions of glycerin was prepared, the viscosities of which could be determined by a commercial viscometer.

Droplet Experiment


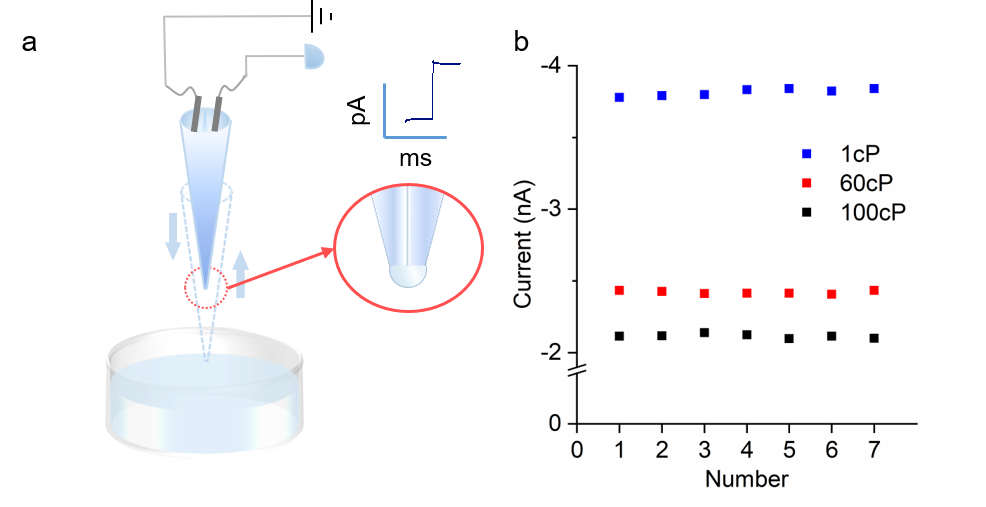


Figure S3**.** a) Experimental setup of the small droplet detection. b) Corresponding current signals of small droplet detection in 1 cP, 60 cP and 100 cP solutions.

As shown in Figure S3a, backfilled with the same detection solution, the viscometer was then immersed into the detection solution and then raised vertically and hold in air, leading to the formation of a small droplet hanging at the nanotip of the viscometer for measuring its viscosity.^[4]^ As recorded in Figure S3b, in the respective seven repeated measurement under the same conditions in 1 cP, 60 cP and 100 cP solutions, the viscometer could recorded rather consistent signals, confirming not only the capability of the nanotool to distinguish different viscosities but also the stability of the viscometer during the repeated measurements.

In Vivo Spatial-resolved Measurements


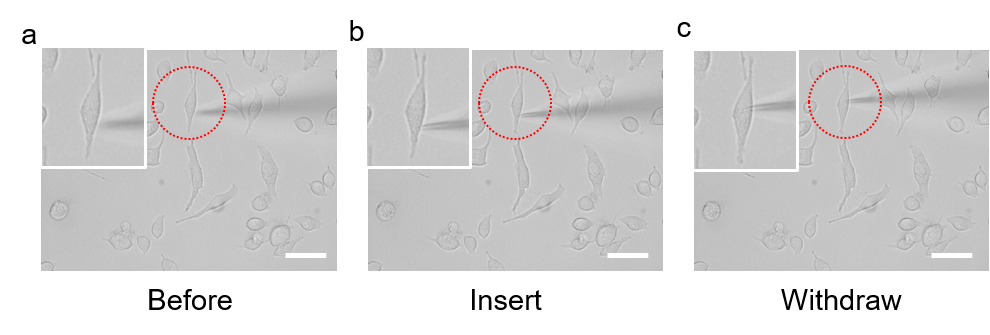


Figure S4**.** Bright-field images of A549 cells before and after penetration and withdrawal of the nanopipette. Scale bar: 50 μm.


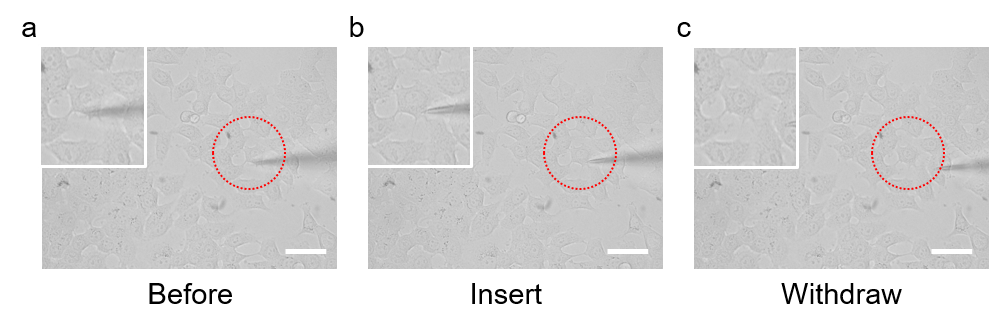


Figure S5**.** Bright-field images of MCF-7 cells before and after penetration and withdrawal of the nanopipette. Scale bar: 50 μm.


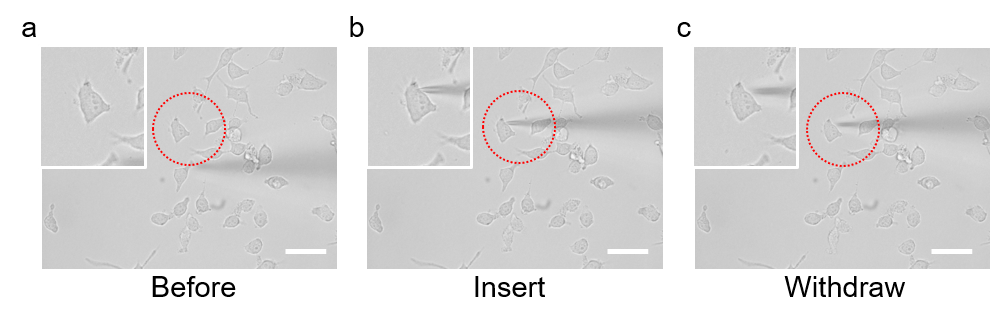


Figure S6**.** Bright-field images of HeLa cells before and after penetration and withdrawal of the nanopipette. Scale bar: 50 μm.

With the assistance of three-dimensional MP-225 micromanipulator, the nanotool could be controlled precisely to insert into specific no A549 (Figure S4), MCF-7 (Figure S5) or HeLa (Figure S6) cells cell without inducing the change of the cellular morphologies.

Cytomembrane Integrity and Cellular Viability


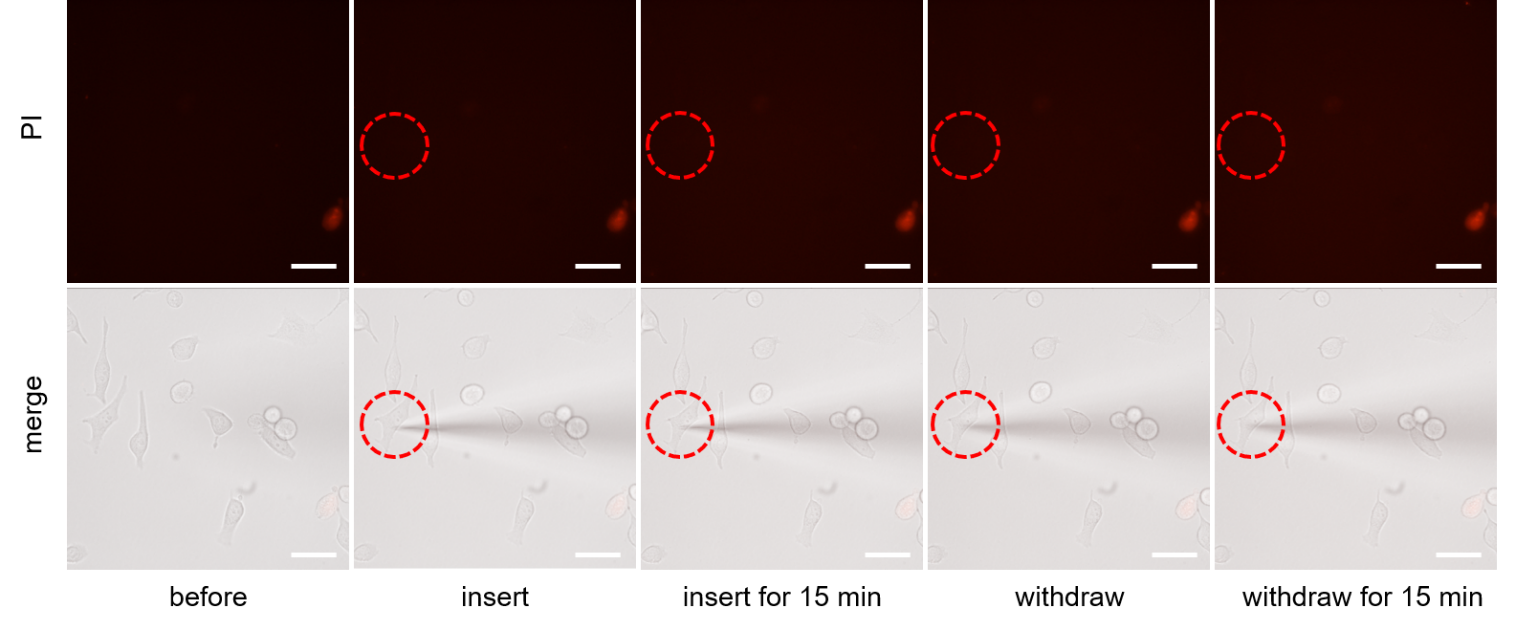


Figure S7**.** Fluorescent and merged images of PI stained HeLa cells before and after the penetration and withdrawal of the nanotool. Scale bar: 50 μm.


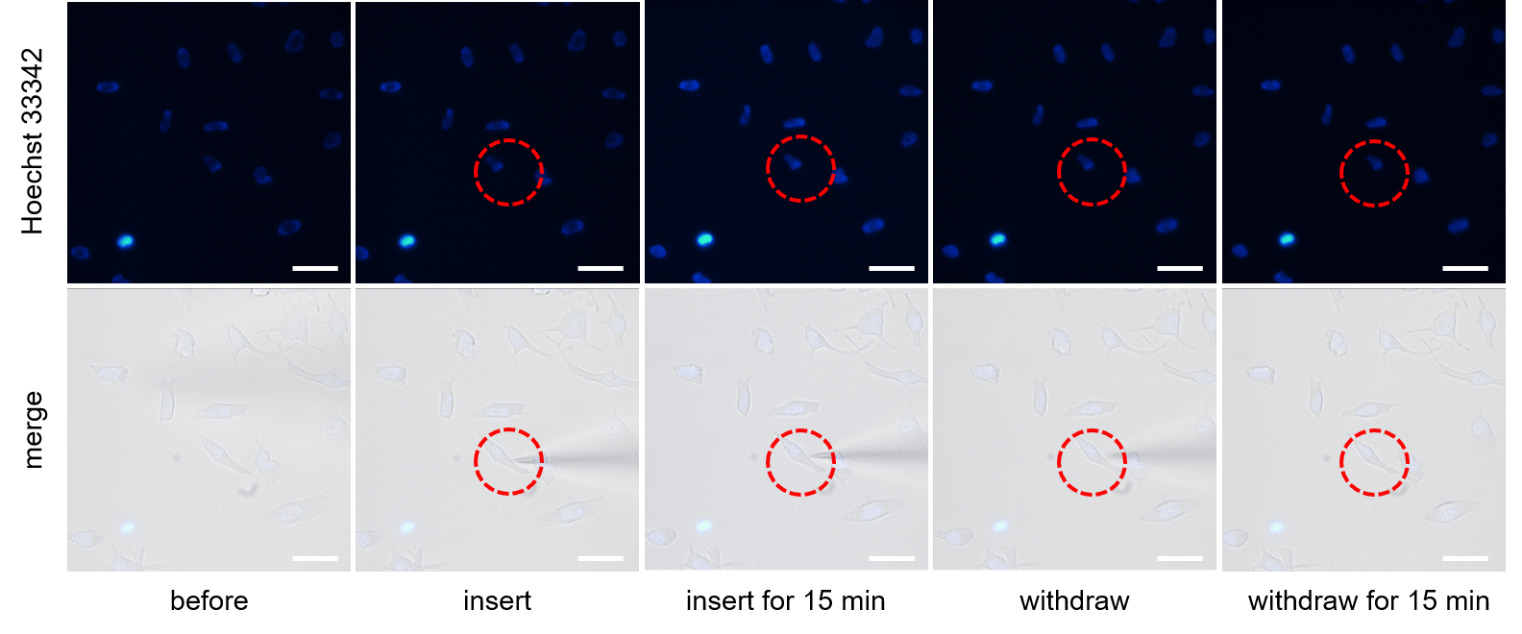


Figure S8**.** Fluorescent and merged images of Hoechst 33342 stained HeLa cells before and after the penetration and withdrawal of the nanotool. Scale bar: 50 μm.


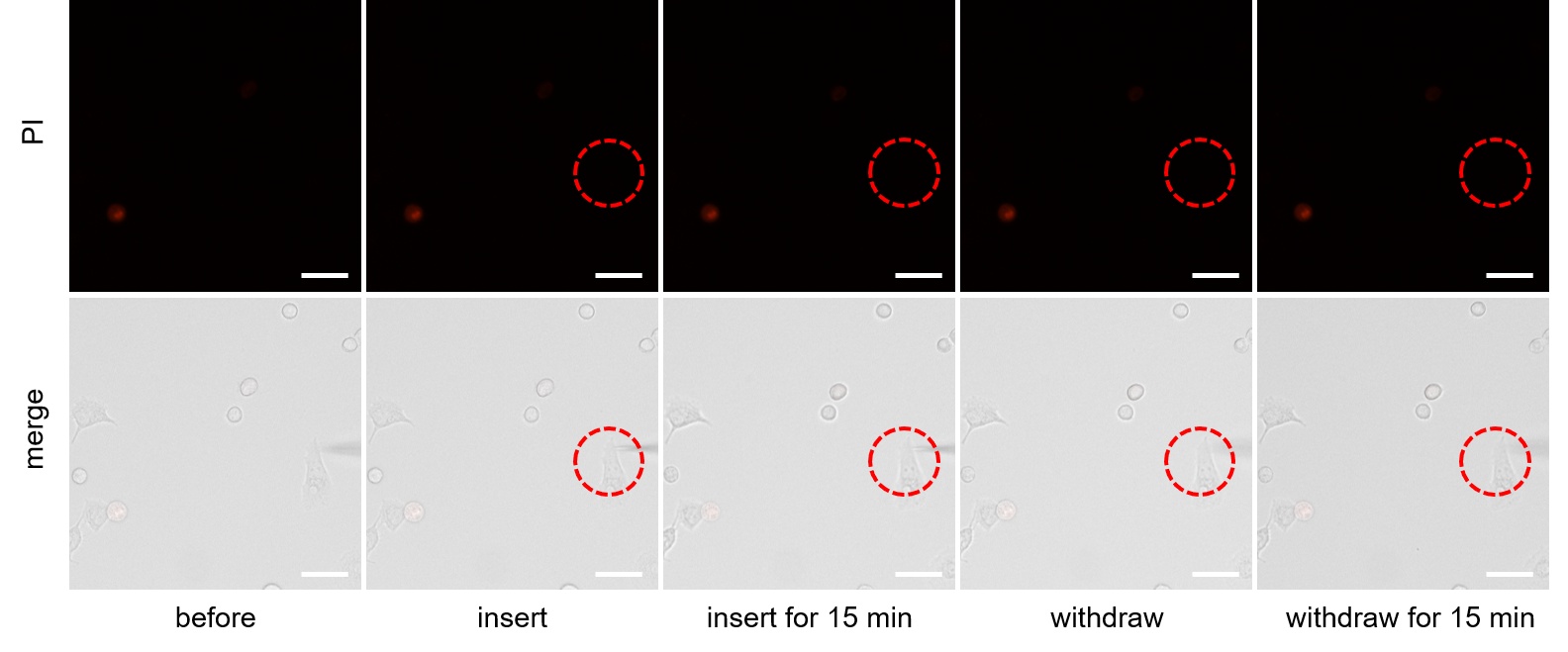


Figure S9**.** Fluorescent and merged images of PI stained MCF-10A cells before and after the penetration and withdrawal of the nanotool. Scale bar: 25 μm.


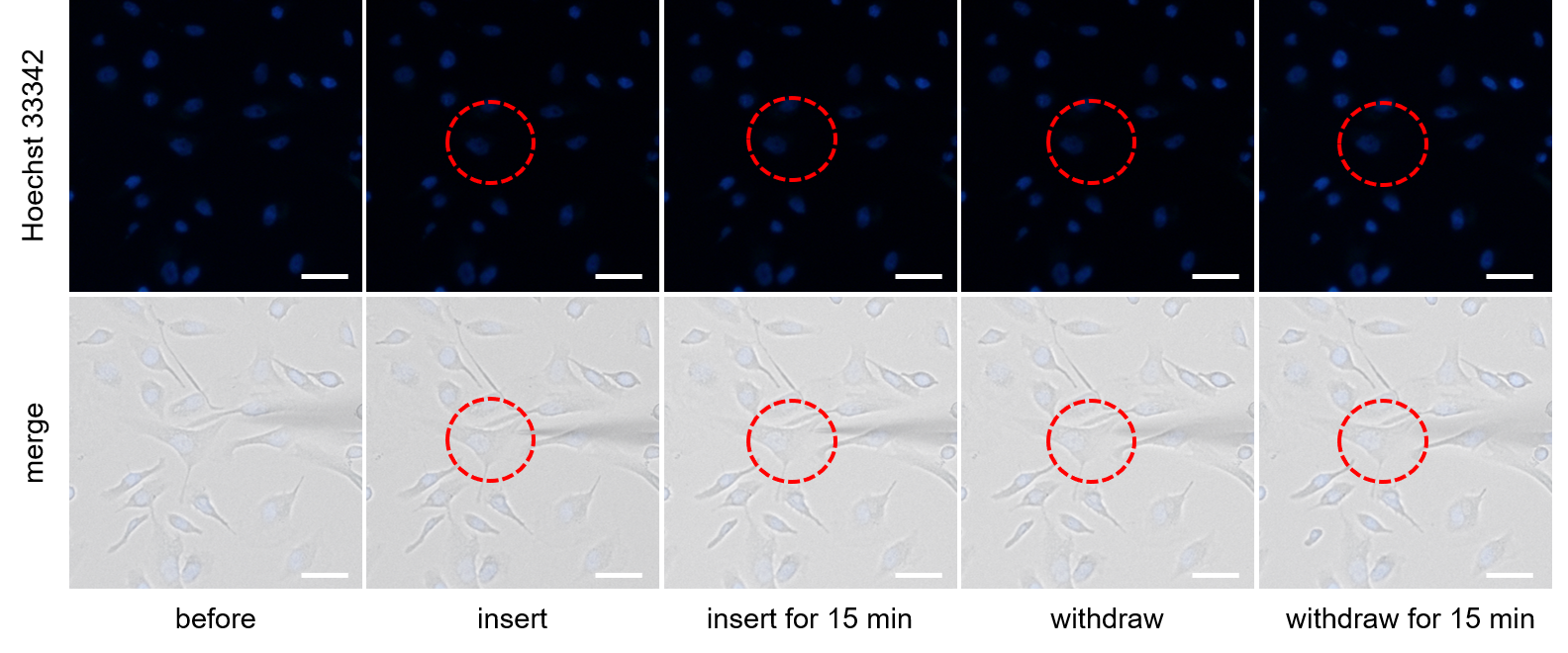


Figure S10**.** Fluorescent and merged images of Hoechst 33342 stained MCF-10A cells before and after the penetration and withdrawal of the nanotool. Scale bar: 25 μm.

To verify the membrane integrity and the cell viability of the cell after insertion, the membrane impermeable dye propidium iodide (PI) and Hoechst 33342 were used to stain the HeLa cells and MCF-10A cells.

PI dye has no membrane impermeability with living cells, but can stain the dead cells to show red fluorescence. The fluorescence micrograph of PI stained HeLa cells before penetration and after withdrawal of the nanotool were shown in Figure S7. After the 15 min of penetration and 15 min of withdrawal, no red fluorescence was observed, indicating the good membrane integrity of the targeted HeLa cell after the penetration of the nanotool.

Hoechst 33342 has a certain membrane permeability and the normal cell nucleuses could be slightly stained and show low blue fluorescence, but the dead cells could show brighter blue fluorescence. The fluorescence micrograph of Hoechst 33342 stained HeLa cells before penetration and after withdrawal of the nanotool were shown in Figure S8. After the 15 min of penetration and 15 min of withdrawal, no change of the fluorescence could be observed, indicating the good cell viability of the targeted HeLa-cell after the penetration of the nanotool.

Incidentally, as shown in Figure S9-S10, the same conclusion has also been proved with noncancerous MCF-10A cell (human mammary epithelial cells).

In Vivo Spatial-resolved Location


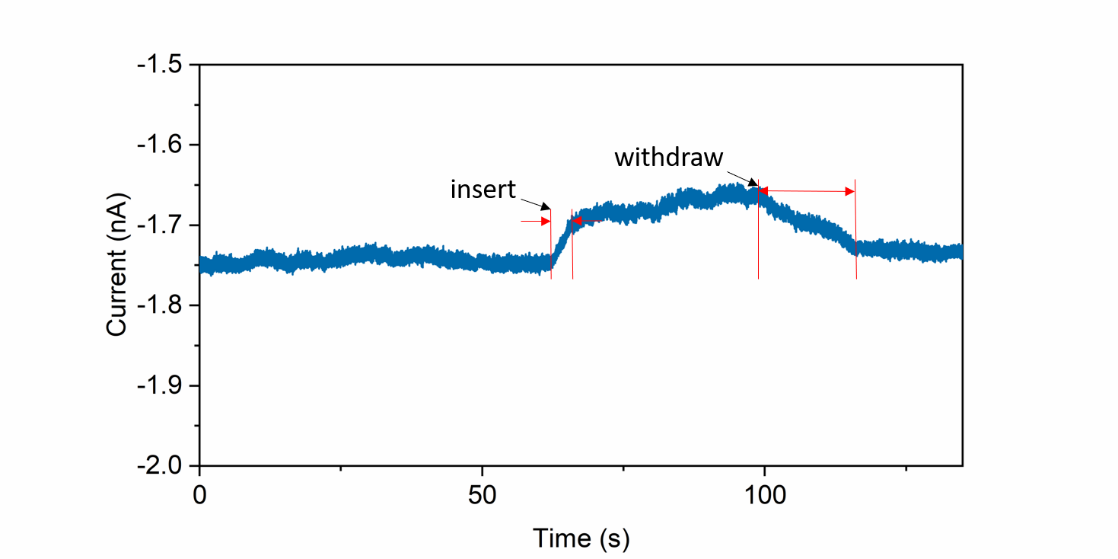


**Figure S11.** The current change of the nanotool during its inserting and withdrawing from the cell membrane at - 200 mV.


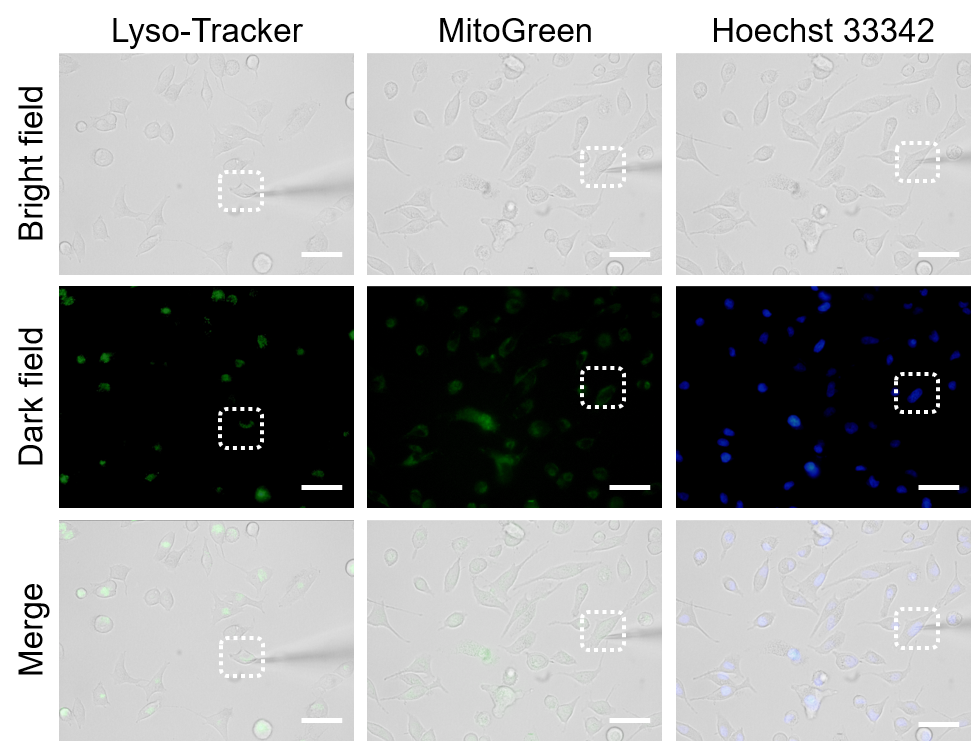


Figure S12**.** In vivo spatial-resolved location of the mitochondrion-dense, lysosome-dense and near nuclear regions of specific A549 cells with the assistance of Lyso-Tracker Green, MitoGreen and Hoechst 33342 dyes. Scale bar: 50 μm.


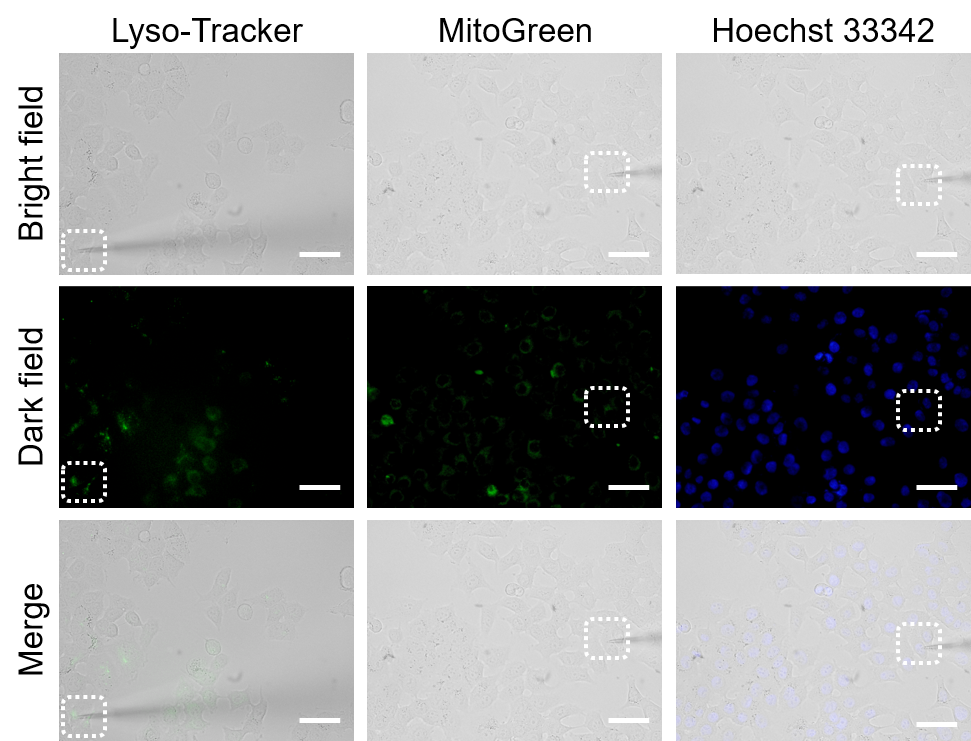


Figure S13**.** In vivo spatial-resolved location of the mitochondrion-dense, lysosome-dense and near nuclear regions of specific MCF-7 cells with the assistance of Lyso-Tracker Green, MitoGreen and Hoechst 33342 dyes. Scale bar: 50 μm.


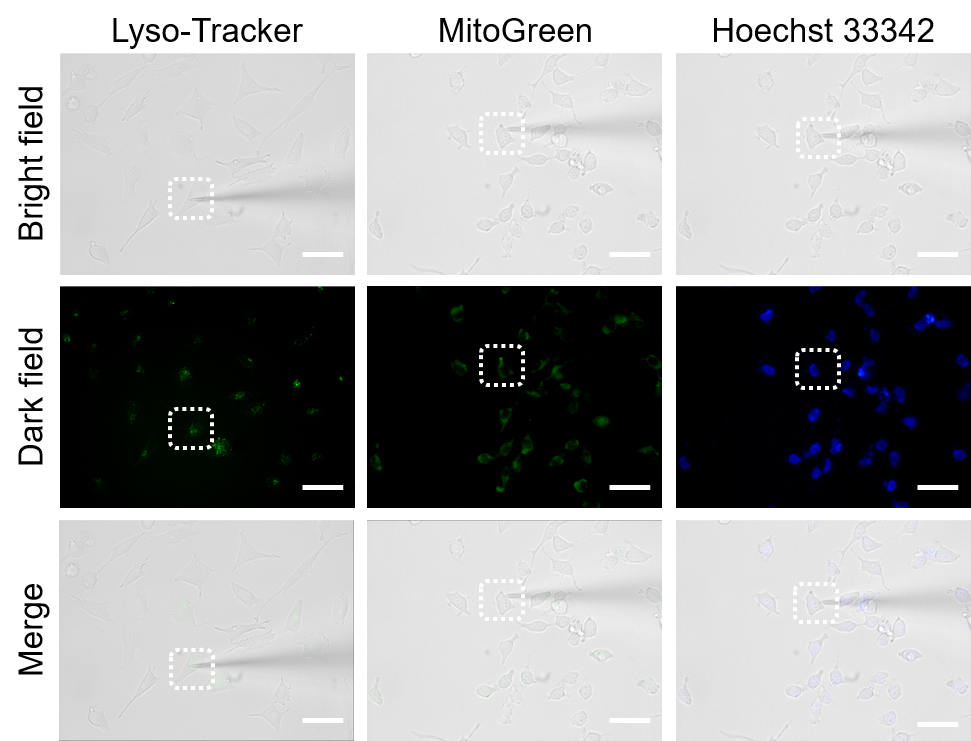


Figure S14**.** In vivo spatial-resolved location of the mitochondrion-dense, lysosome-dense and near nuclear regions of specific HeLa cells with the assistance of Lyso-Tracker Green, MitoGreen and Hoechst 33342 dyes. Scale bar: 50 μm.





Figure S15. The corresponding ionic currents collected at lysosomes-dense, mitochondria-dense, and near-nucleus regions (n=25).

Figure S15 showed the corresponding current during the nanotip penetrated throughout the cell membrane. With the assistance of Lyso-Tracker Green, MitoGreen and Hoechst 33342, lysosomes-dense, mitochondria-dense and near nuclear regions were respectively located in A549 (Figure S14), MCF-7 (Figure S15) and HeLa (Figure S16) cells. The corresponding ionic currents in these locations were recorded as shown in Figure S17.

Glucose Deprivation Detection





Figure S16**.** Viscosity of normal HeLa cell within 60 minutes.

Targeting HeLa cells, the viscous change around ca. 60 cP of which was monitored as shown in Figure S16.

Characterization of the Formation of Intracellular Stress Granules





Figure S17**.** Fluorescence microscopy images of TIA-1 marked normal, heat shocked and recovered HeLa cells after 60 minutes recovery. Scale bar: 10 μm.

Figure S17 showed the progress of the SGs (white arrow) assembly as well as disassembly, as indicated by the small particles exhibiting strong green fluorescence.





Figure S18**.** a) Bright-field microscopy images of the nanotool recording the ionic signals within the single HeLa cell upon heat-shock recovery experiment. Scale bar = 10 μm.

Figure S18 showed the targeted heat-shock recovery cell well maintained its morphology during the 60 min detection.


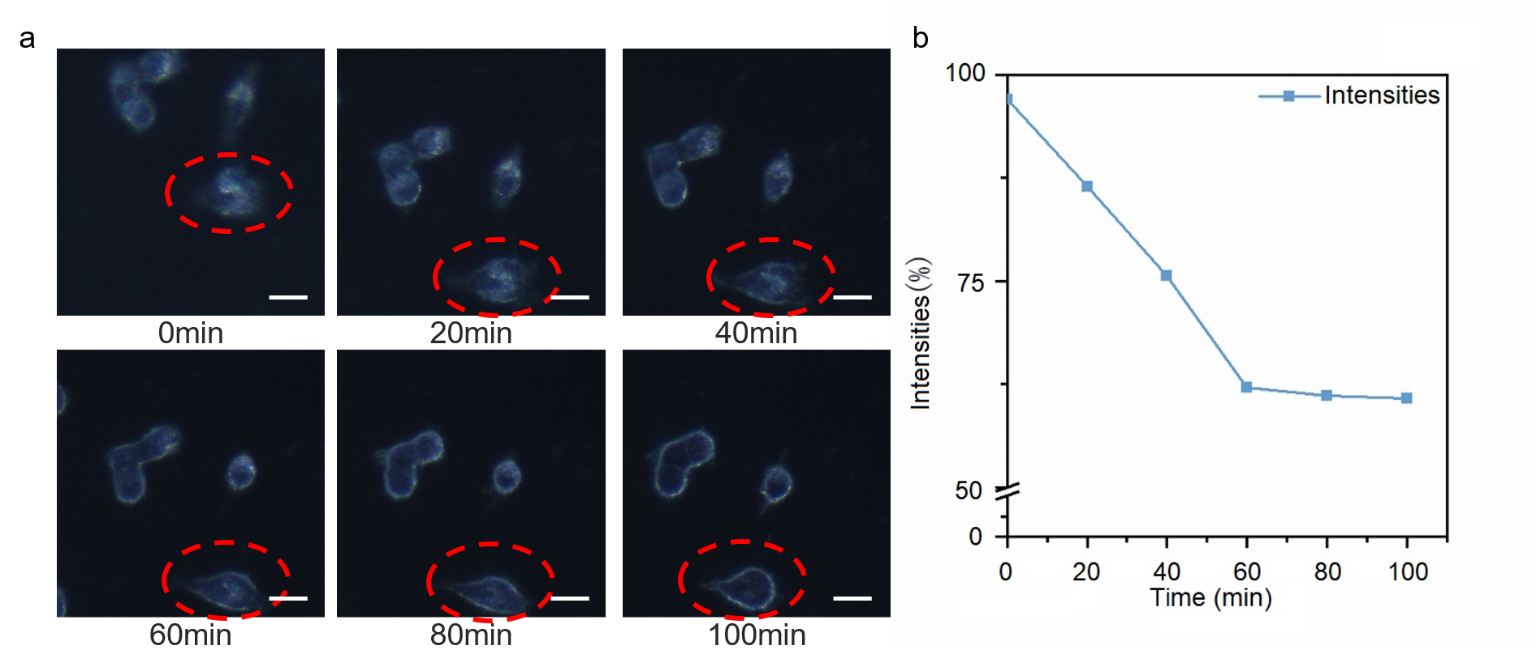


Figure S19**.** a) Dark field images of the heat-shocked HeLa cell during 100 minutes observation; b) The corresponding change of the light intensities. Scale bar = 20 μm.

Figure S19a shows the HeLa cell brightness of the heat-shocked HeLa cell decreased with time during 100 min observation, indicating the disassembly of SGs. Figure S19b shows the corresponding brightness analysis processed by imaged J software.

Some Typical Applications of θ-nanopipettes in Bioanalysis and Beyond

Table S2. Some typical applications of θ-nanopipette in bioanalysis and beyond.

| Experiment method | Descriptions | Applications of analysis | Ref. |
| --- | --- | --- | --- |
| Microdroplet fusion mass spectrometry | Electrogenerating and catching one reactive intermediate | Identifying labile intermediates | [5] |
| Dielectrophoretic (DEP) | Trapping molecules and controlling active analyte transportation as well as pre-concentration | Detecting single molecules in solution environments | [6] |
| Minimally invasive nanotweezers | Extract material with low invasive damage | Single molecule analysis in living cell | [7] |
| Scanning Ion Conductance Microscopy (SICM) | Self-assembly functionalized hydrogel detecting extracellular pH (pHe) with high-resolution | Local pHe detection around a single cell | [8] |
| Molecule device | Actively controlling DNA transport and efficiently bridging molecules between two pores with high resolution | Controlling DNA transport and efficiently bridging molecules between two pores | [9] |
| Nanopore extended field-effect transistor | Switch on/off, and slow down single-molecule DNA transport via controllable gate voltage | Detecting single-molecule translocation events of DNA and IgG antibodies | [10] |
| Electroosmosis | A barrel is used to collect cytosol and the other served as SICM imaging | Collecting cytosol from living cells | [11] |
| SECM-SICM | Double-barrel carbon nanoprobes combined with SICM distance feedback control | Localized chemical stimulation and detection of cellular neurotransmitter release | [12] |

References

[1] Becker, Stephen R., Peter H. Poole, and Francis W. Starr. "Fractional Stokes-Einstein and Debye-Stokes-Einstein Relations in a Network-Forming Liquid," *Physical Review Letters*, vol. 97, no. 5, article 55901, 2006.

[2] G. J. Hirasaki, S. W. Lo and Y. Zhang, "NMR Properties of Petroleum Reservoir Fluids." *Magnetic Resonance Imaging*, vol. 21, no. 34, pp. 269-277, 2003.

[3] S. A. Martemyanov, N. V. Petrovskiy and B. M. Grafov, "Turbulent Pulsations of the Microelectrode Limiting Diffusion Current.," *JOURNAL OF APPLIED ELECTROCHEMISTRY*, vol. 21, pp. 1099-1102, 1991.

[4] P. Cadinu, B. Paulose Nadappuram, D. J. Lee et al., "Single Molecule Trapping and Sensing Using Dual Nanopores Separated by a Zeptoliter Nanobridge," *Nano Letters*, vol. 17, no. 10, pp. 6376-6384, 2017.

[5] J. Hu, T. Wang, W. J. Zhang et al., "Dissecting the Flash Chemistry of Electrogenerated Reactive Intermediates by Microdroplet Fusion Mass Spectrometry," *Angewandte Chemie International Edition*, vol. 60, no. 34, pp. 18494-18498, 2021.

[6] L. Tang, B. P. Nadappuram, P. Cadinu et al., "Combined Quantum Tunnelling and Dielectrophoretic Trapping for Molecular Analysis at Ultra-Low Analyte Concentrations," *Nature Communications*, vol. 12, no. 1, pp. 913-921, 2021.

[7] B. P. Nadappuram, P. Cadinu, A. Barik et al., "Nanoscale Tweezers for Single-Cell Biopsies," *Nature Nanotechnology*, vol. 14, no. 1, pp. 80-88, 2019.

[8] Y. Zhang, Y. Takahashi, S. P. Hong et al., "High-Resolution Label-Free 3D Mapping of Extracellular pH of Single Living Cells," *Nature Communications*, vol. 10, no. 1, article 5610, 2019.

[9] P. Cadinu, G. Campolo, S. Pud et al., "Double Barrel Nanopores as a New Tool for Controlling Single-Molecule Transport," *Nano Letters*, vol. 18, no. 4, pp. 2738-2745, 2018.

[10] R. Ren, Y. Zhang, B. P. Nadappuram et al., "Nanopore Extended Field-Effect Transistor for Selective Single-Molecule Biosensing," *Nature Communications*, vol. 8, no. 1, pp. 586-594, 2017.

[11] Y. Nashimoto, Y. Takahashi, Y. Zhou et al., "Evaluation of mRNA Localization Using Double Barrel Scanning Ion Conductance Microscopy," ACS Nano, vol. 10, no. 7, pp. 6915-6922, 2016.

[12] Y. Takahashi, A. I. Shevchuk, P. Novak et al., "Multifunctional Nanoprobes for Nanoscale Chemical Imaging and Localized Chemical Delivery at Surfaces and Interfaces," *Angewandte Chemie International Edition*, vol. 50, no. 41, pp. 9638-9642, 2011.
